# Can Amlodipine Improve the Pre-ovulatory Follicle Blood Flow in Women with Polycystic Ovarian Syndrome?

**Published:** 2019

**Authors:** Doaa El Faham, Khaled Ali, Adel Salah El Din, Mamdouh Bibars, Osama Azmy

**Affiliations:** 1. Reproductive Health Research Department, National Research Centre, Cairo, Egypt; 2. Obstetrics and Gynecology Department, Ain Shams University Maternity Hospital, Cairo, Egypt

**Keywords:** Amlodipine, Clomiphene Citrate, Doppler ultrasonography, Infertility, Pre-ovulatory follicle

## Abstract

**Background:**

A reduction in intra-ovarian vascular resistance is necessary to achieve pregnancy in a natural cycle. The aim of this RCT was to detect whether a vasodilator calcium channel blocker, amlodipine, could increase the pre-ovulatory follicular blood flow, enhance follicular maturation in women with PCOS and improve ovulatory outcome.

**Methods:**

Sixty women received induction by clomiphene citrate (CC); thirty were given amlodipine (Amlodipine group) and the other 30 women were given placebo (Placebo group). The pattern of pre-ovulatory follicle blood flow was studied by color and power Doppler ultrasonography pre and post drug administration. Independent t-test was used to compare mean values of the 2 groups. The p<0.05 is considered statistically significant.

**Results:**

When comparing the Doppler effect of amlodipine versus placebo in the treatment cycle, it was found that mean value of ovarian arteries (OA) pulsatility index was lower in amlodipine group but it didn’t reach statistical significance (p= 0.063); however, the mean value of OA resistance index reached statistical significance (p=0.028) in amlodipine group. Moreover, in the second cycle, endometrial thickness was significantly higher (p=0.006) in women of the amlodipine group when compared to those of the placebo group. At least one sonographically detectable mature follicle (≥18 *mm*) was observed in 54.5% (36/66) during the first cycle. At the second cycle, this proportion significantly rose to 86.7% (26/30) in the amlodipine group, but marginally and non-significantly to 56.7% (17/30) in the placebo group.

**Conclusion:**

Amlodipine as calcium channel blocker was proved to have a role in improving ovarian blood flow at the time of ovulation and enhancing follicular maturation and thus, it may increase the chances of conception.

## Introduction

Polycystic ovarian syndrome (PCOS) is a very common endocrine disorder (1). Infertility in women with PCOS is due to oligo- or anovulation. Several theories have been proposed to explain anovulation in PCOS women; one of these theories showed that follicular unresponsiveness is due to Insulin Resistance (IR) and compensatory hyperinsulinemia (2). It was found that IR occurs in the women with PCOS even if oral glucose tolerance test was normal and they have an increased risk of premature atherosclerosis and metabolic syndrome (3). Also, excess androgens in PCOS women have a direct vasoconstrictive effects on vascular tissues (4), thereby increasing vascular resistance. Therefore, PCOS women have an increased predisposition to atherosclerosis with resultant hardening and thickening of vessel walls (5), which may also result in increased systemic vascular resistance. Presumably diminished uterine blood flow seems to be one of the principal problems in the pathogenesis of infertility or sub-fertility in women with PCOS who have hyper-androgenism or are obese (6), and may be one of the mechanisms by which these women have reduced fecundity and an increased risk of aberrant pregnancy outcome (7, 8). Several studies and clinical trials introduced new therapies in this area and their main action was directed to increase the ovarian, endometrial and subendometrial blood flow aiming to enhance the process of ovulation, improve ovum quality and increase endometrial receptivity. Some studies have shown that nitric oxide (NO) plays a role in the mechanisms of ovulation, corpus luteum formation and implantation (9, 10). Others stated that vitamin E, L-arginine, or sildenafil citrate treatment improves uterine radial artery-RI and endometrial thickness and may be useful for the patients with a thin endometrium (11). The aim of this multicenter randomized controlled trial was to detect whether a vasodilator calcium channel blocker, amlodipine, could normalize the pre-ovulatory follicular blood flow and enhance follicular maturation in women with PCOS.

## Methods

**Figure 1.**
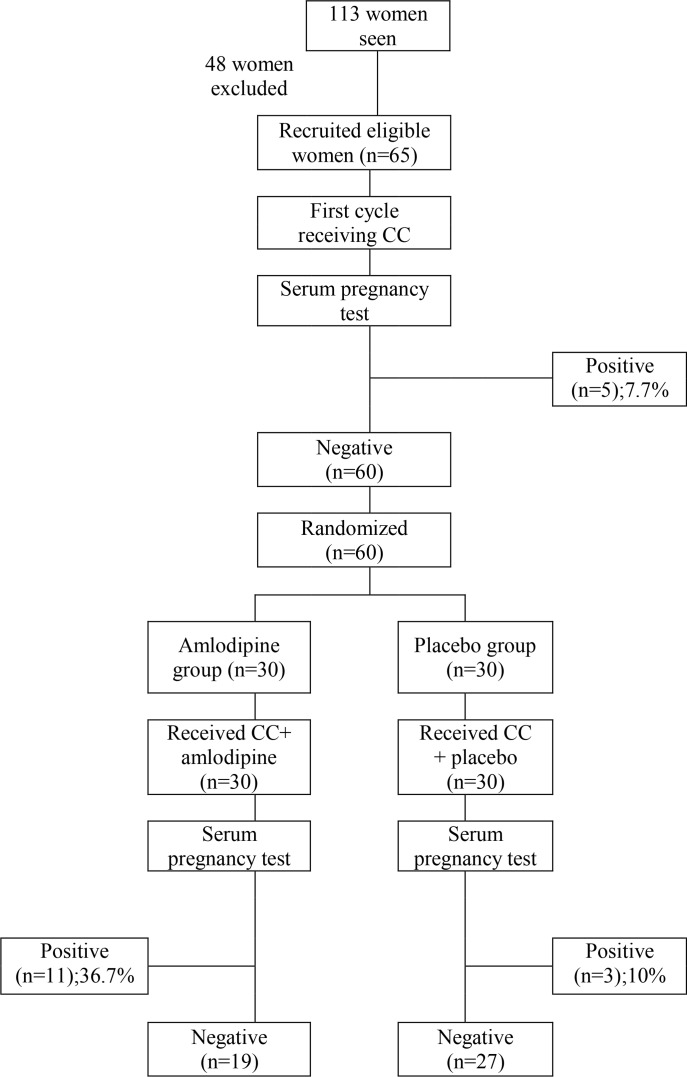
Flow diagram showing study course

This trial was conducted at the infertility clinics of Medical Research Centre of Excellence at the National Research Centre, Ain Shams University Maternity Hospital. It was conducted during the period from February to September 2013. The study was approved by Ethical Committee of the National Research Centre and registered with the Pan African Clinical Trial Registry. A total of 113 infertile women were seen and examined. Only sixty five infertile women with PCOS were recruited (Figure 1) with the inclusion criteria of age of ≥20 and ≤39 years, and body mass index (BMI) of ≤40 *kg/m*^2^, infertility secondary to PCOS, as indicated by presence of at least two of the following criteria according to The Rotterdam consensus workshop (12); clinical hyperandrogenism (Ferriman Gallwey score>8) or biochemical hyperandrogenism (Elevated total/free testosterone), oligomenorrhea or oligo-ovulation, polycystic ovaries on ultrasound (≥12 follicles in one ovary or ovarian volume ≥10 *cm*^3^). Women were excluded from the study if they had other causes of infertility diagnosed during infertility workup; if they got pregnant, if they received more than 4 cycles of CC or any form of contraception in the previous year from recruitment. Hepatic, cardiac, and hypotensive (Blood pressure <90/60 *mm/Hg*) women were also ruled out from the study.

Clomiphen citrate was used for ovulation induction for both the amlodipine and placebo groups. A 100 *mg* single morning dose of clomiphene citrate (Clomid, Sanofi Aventis, Paris, France) was given for 5 days starting from the second day of the menstrual cycle according to the recommendation of the manufacturer (13). Before embarking on giving amlodipine in the second cycle, randomization started. CC as the previous dosage and amlodipine 5 *mg* (Norvasc, Pfizer Inc, Egypt) were given orally in the second cycle. Amlodipine was given from day 6 to day 12 of the cycle once per day just before bed time to minimize the side effects. In this study, amlodipine was selected because it inhibits calcium ion influx across cell membranes selectively, with a greater effect on vascular smooth muscle cells than on cardiac muscle cells (14). This action is of gradual onset and long half life (half life is from 35 to 45 *hr*) (15). Moreover, it is a cheap drug with the least side effects; swelling of legs, tiredness or sleepiness, dizziness, palpitations, arrhythmia are the typical side effects and they were surpassed by giving the drug at bed time. 5mg dose was given and normotensive non-cardiac patients were included.

### Study design and randomization:

This was a double-blind prospective randomized controlled interventional trial. Randomization started after the first cycle. The placebo was a tablet made from inert excipient (USP grades) as hydroxypropyl methyl cellulose, direct compression was done to a flat-faced round punches of 6 *mm* diameter using single punch tablet machine (Stokes-Merril-model 511-7-A). The placebo was manufactured by the department of industrial pharmaceutics at the National Research Centre to mimic the shape and size of the drug tablet. Women were blinded by putting the drug or the placebo inside a small opaque sealed inert container (Bottle) manufactured by the industrial department of the National Research Centre; the drug had letter A and the placebo had letter B on the bottle. An informed consent explaining the clinical trial and possible side effects of amlodipine was obtained from each woman prior to recruitment. Women fulfilled the inclusion criteria were randomized using a computer based allocation. Distribution concealment was attained using closed opaque and sealed envelope method.

### Ultrasonography:

Ultrasonography, power and color Doppler analysis were performed for two consecutive cycles. This was done using Voluson E6 (GE Healthcare,Wisconsin, USA). The expected time for ovulation was determined by folliculometry which started on day 9 and women were followed up every other day till day 13 of the cycle. Women were left for spontaneous ovulation without human chorionic gonadotropin administration. The association between the administered medications and the increase in blood flow to the preovulatory follicle was assessed using power and color Doppler analysis by the same ultrasonographer. The pulsatility and resistance indices (PI, RI), and peak systolic velocity of ovarian, follicular and uterine blood flow were automatically calculated, the size of dominant follicles and endometrial thickness were measured on day 13 of the cycle. No correction was made for the angle of insonation of the Doppler beam for ovarian arteries. Color Doppler allows for rapid orientation and localization of the vessels. Blood flow velocity waveforms were recorded with the sample volume placed across the vessel and the pulsed Doppler mode entered. The measurements were done twice and the average of the recorded readings was taken.

### Statistical analysis:

Quantitative data were proved for normality by Shapiro-Wilk test. Independent t-test was used to compare mean values of the 2 groups. Chi square test was used to compare between percentages of the two groups. The p<0.05 was considered statistically significant (SPSS V. 22.1, Microsoft, California, USA).

## Results

There were no significant differences between women of both groups regarding their age or body mass index (BMI). The difference between the pulsatility index (PI) and resistance index (RI) of ovarian arteries was statistically non-significant in the first cycle. During the second cycle, mean value of ovarian arteries (OA) and pulsatility index were lower in amlodipine group but it didn’t reach statistical significance (1.16±0.87 *vs.* 1.60± 0.93 p=0.063); however, the mean value of OA resistance index reached statistical significantly (0.61±0.18 *vs.*0.73±0.23; p=0.028) in amlodipine group when compared to placebo group (Tables 1 and 2). Additionally, on assessing the mean endometrial thickness of both studied groups, on the day of detecting a mature follicle in the first cycle, it was statistically non-significant (7.33±2.13 *vs.* 7.31±2.12), while in the second cycle, it was significantly higher (p=0.006) in women of the amlodipine group (9.2±2.1 *mm*) when compared to those of the placebo group (7.4±2.4 *mm*).

**Table 1.** Comparison between the groups in the ovulation cycle before and after the intervention

	**Amlodipine group Mean±SD (n=32)**	**Placebo group Mean±SD (n=33)**	p-value^*^
**Age (years)**	27.2±3.6	26.7±5.3	0.633
**BMI (*kg/m*^2^)**	27.8±3.7	28.1±4.2	0.548
**Ovulation induction cycle before the intervention**			
**Ovarian artery PI**	1.55±0.87	1.58±1.10	0.90
**Ovarian artery RI**	0.71±0.16	0.69±0.18	0.65
**Endometrial thickness (*mm*)**	7.33±2.13	7.31±2.12	0.971
	N (%)	N (%)	RR (95% CI)
**Follicle ≥ 18 *mm* on ultrasound**	16/32 (50.0%)	17/33 (51.5%)	0.79
**Clinical pregnancy**	3/32 (9.4%)	2/33 (6.1%)	0.32
**Ovulation induction cycle after the intervention**			
	**Mean±SD (n=30)**	**Mean±SD (n=30)**	
**Ovarian artery PI**	1.16±0.87	1.60±0.93	0.063
**Ovarian artery RI**	0.61±0.18	0.73±0.23	0.028
**Endometrial thickness (*mm*)**	9.2±2.07	7.41±2.42	0.006
	N (%)	N (%)	RR (95% CI)
**Follicle ≥18 *mm* on ultrasound**	26/30 (86.7%)	17/30 (56.7%)	2.57 (1.06–6.26)
**Clinical pregnancy**	11/30 (36.7%)	3/30 (10%)	1.90 (1.23–2.95)

Induction of ovulation was performed using CC 100 *mg/day* during 5 days in both groups.

* p-value was determined by unpaired t test

**Table 2.** Comparison between the groups in the ovulation cycle before and after the intervention

	**Amlodipine group**		p-value ^*^	**Placebo group**		p-value ^*^
	
	**n=30** **Mean±SD**	**n=30** **Mean±SD**		**n=30** **Mean±SD**	**n=30** **Mean±SD**	
	
	**Before**	**After**		**Before**	**After**	
**Ovarian artery PI**	1.51±0.77	1.16±0.87	0.035	1.51±1.08	1.60 ± 0.93	0.656
**Ovarian artery RI**	0.73±0.15	0.61±0.18	0.001	0.67±0.2	0.73±0.23	0.167
**Endometrial thickness (*mm*)**	7.12±2.01	9.2±2.07	<0.001	7.28±2.10	7.41 ± 2.42	0.774

Induction of ovulation was performed using CC 100 *mg/day* during 5 days in both groups.

* p-value was determined by paired t test

In the first cycle, at least one sonographically-detectable mature follicle (≥18 *mm* in average dimension) was observed in 55.4% (36/65). During the second cycle, this proportion significantly rose to 86.7% (26/30) in the amlodipine group, but marginally and non-significantly rose to 56.7% (17/30) in the placebo group. The difference between the two groups in the second cycle regarding the rate of detection of at least one mature follicle was significant in favor of amlodipine group.

During the first cycle, four women out of 65 (6.2%) had more than one sonographically-detectable mature follicle, whereas, this proportion significantly rose to 23.3% (7/30) in the amlodipine group, and significantly rose to 30% (10/30) in the placebo group during the second cycle. Administration of amlodipine was associated with a 30% increased rate of having at least one mature follicle [NNT≅3] (Table 1).

Regarding the pregnancy rate, five women (7.7%) got pregnant during the first cycle and they were excluded from the study. In the second cycle, this proportion significantly rose to 36.7% (11/30) in the amlodipine group, but marginally and not significantly to just 10% (3/30) in the placebo group. The difference between the two groups in the second cycle regarding the pregnancy rate was significant and in favor of the amlodipine group with a 26.7% higher pregnancy rate [NNT≅4].

## Discussion

This study showed that the amlodipine as a calcium channel blocker improved ovarian blood flow with subsequent improvement in ovulation and pregnancy rates. Amlodipine balances the blood flow between the ovaries and uterus, it decreases the peripheral resistance and tends to normalize ovarian and uterine blood flow; this was reflected by both ovarian vessels Doppler indices and the sizes of mature follicles in amlodipine group. Additionally, the increase in endometrial thickness on day 13 of the cycle confirms this finding. The improvement in the ovarian blood flow and the size of the dominant follicles, which have occurred after the amlodipine administration, could be attributed to eliminating the direct vasoconstrictive effects of excessive androgen present in PCOS cases.

Amlodipine was given after the selection of the dominant follicles in previously primed ovaries by CC. Giving amlodipine before induction and selection could have aggravated and worsen the pathogenesis of polycystic ovarian pattern, as this could have increased the blood supply to the cohort of follicles in PCOS women.

The induction with CC for two consecutive cycles seems to have a cumulative effect through finding higher rate of detecting more than one mature follicle. However, administration of amlodipine seems to reduce this risk from 30% to 23.3%, *i.e*. by 6.7%, NNT≅15. One limitation of our study was that the sub-endometrial blood flow was not included in the data collected and only endometrial thickness was measured. Therefore, assessment of the sub-endometrial blood flow in future studies would be a good strategy to evaluate the real effect of the drug in enhancing receptivity through increasing the blood supply to the endometrium.

It is assumed that adding a vasodilator drug to the gold standard ovulation induction drugs may improve the clinical pregnancy rate not only the ovulation rate. This could be due to avoidance of the deleterious effect of the thin endometrium induced by the anti-estrogenic drugs on implantation. Vaginal sildenafil, for example, combined with ovarian stimulation was shown to improve endometrial thickness from 8 to 10 *mm* and three of the four women became pregnant (17).

Sildenafil has a comparable effect to amlodipine, but it is more expensive with more side effects. It has significant effect on pregnancy rate per cycle, as three out of four women became pregnant after sildenafil, but it was tested only on its effect on the endometrium and not on the ovarian blood flow or the size and number of the dominant follicles.

Nitric oxide has been found to be an important regulator of vascular resistance (18, 19 and 20) and for this reason was investigated for its possible role in the regulation of ovarian hemodynamics at ovulation and its possible effects on the blood-follicle barrier. Studies have shown that NO plays a role in the mechanisms of ovulation, corpus luteum formation and implantation (9).

Using pentoxifylline and vitamin E may improve the pregnancy rate in women with a thin endometrium by increasing the endometrial thickness (21). Comparative studies are required to assess the effect of pentoxifylline and vitamin E improving endometrial thickness and pregnancy rate as compared to amlodipine.

From the current study, it has been proposed that the mechanism of action of amlodipine was decreasing the peripheral resistance in order to increase the blood flow to the pre-ovulatory follicles and thus increasing the level of LH hormone in the ovary. This occurred after the recruitment of the dominant follicle. By stopping amlodipine, there would be a decrease in the blood supply of the apical area of the follicle occurring 45 to 72 *hr* later. This apical ischemia would facilitate the process of ovulation and would be entirely mimicking the physiological pattern. Furthermore, amlodipine had an additional effect of increasing endometrial thickness.

## Conclusion

Amlodipine seems to be a promising drug in improving uterine, ovarian blood flow, size of preovulatory follicle and pregnancy rate in women with PCOS. It was proved in this study that Amlodipine was capable of improving ovarian blood flow and enhancing follicular maturation at the time of ovulation, thus, it may increase the chances of conception.
